# Predictors for vertebral height deterioration in fractured vertebrae operated by percutaneous vertebroplasty

**DOI:** 10.1186/s12891-025-08574-3

**Published:** 2025-04-03

**Authors:** Benqiang Tang, Xueming Chen, Libin Cui, Yanhui Wang, Xin Yuan, Yadong Liu, Liang Liu

**Affiliations:** Department of Orthopedics, Beijing Luhe Hospital, Capital Medical University, No.82, Xinhua South Road, Touzhou District, Beijing, 101149 China

**Keywords:** Osteoporotic vertebral compression fracture, Osteoporosis, Percutaneous vertebroplasty, Vertebral height, Recompression

## Abstract

**Background:**

Vertebral height loss of fractured vertebrae treated by percutaneous vertebral augmentation (PVA) for osteoporotic vertebral compression fracture (OVCFs) during follow-up had been reported. Mostly, vertebral height loss and its relevant terms (e.g., “recompression”, “recollapse” and “refracture”) were defined according to immediate postoperative vertebral height as the baseline in published studies. By contrast, vertebral height deterioration (VHD) was defined according to preoperative vertebral height as the baseline in the present study. The aim of the study was to reveal predictors for VHD in fractured vertebrae operated by percutaneous vertebroplasty (PVP), with a specific focus on surgical factors.

**Methods:**

All patients with OVCFs treated by PVP between April 2016 and September 2018 were retrospectively reviewed. Patients were followed up for at least 12 months after procedure according to treatment protocol. VHD was defined as the presence of a decrease of vertebral height at final follow-up compared to preoperative. Clinical, radiological and surgical factors that might affect occurrence of VHD were assessed using univariate and multivariate analyses.

**Results:**

A total of 543 patients (females 80%, age 73.2 ± 8.1 years) with 681 fractured vertebrae who underwent PVP were enrolled. Mean follow-up time was 28.9 ± 13.4 months (range, 12–59 months). Incidence of VHD in fractured vertebrae was 48.9% (333/681). One clinical factor and four radiological factors, including fracture age (OR = 0.513, 95% CI 0.385–0.683, *p* = 0.000), fracture location (OR = 2.878, 95% CI 1.994–4.152, *p* = 0.000), fracture severity (OR = 0.521, 95% CI 0.386–0.703, *p* = 0.000), cortical defect on lateral wall (OR = 2.535, 95% CI 1.351–4.758, *p* = 0.004) and intravertebral cleft (OR = 2.362, 95% CI 1.488–3.750, *p* = 0.000), were independent predictors for VHD. However, all the surgical factors evaluated were not significant in final model analysis.

**Conclusions:**

Surgical factors might play a negligible effect on VHD. VHD might be due to natural course of fracture/osteoporosis.

## Background

Percutaneous vertebral augmentation (PVA), including percutaneous vertebroplasty (PVP) and percutaneous kyphoplasty (PKP), had been widely accepted as safe and effective treatment option for osteoporotic vertebral compression fracture (OVCFs). For the augmented vertebrae, vertebral height had been paid attention preoperatively, postoperatively and at follow-up [1, 2]. Vertebral height restoration was defined as the differences between postoperative and preoperative. It was a parameter usually indicating the immediate effect of procedure [3]. Vertebral height loss was defined as the changes between at follow-up and immediate postoperative. It was a parameter tentatively reporting the gradual consequences of disease [4, 5]. Based on this meaning of vertebral height loss, some relevant terms in literature, such as “recompression”, “recollapse” or “refracture”, were further defined qualitatively and quantitatively [4–8].

Potential mechanisms underlying vertebral height loss might attribute to the progression of osteoporosis, bone remodeling processes or biomechanical factors (e.g., bending load or static stress). Clinically, in order to provide a better understanding on whether vertebral height loss was procedure-related or part of the natural features of patient/fracture, multivariate analysis [4–9] and pooled analysis [10, 11] had been used to identify risk factors for the occurrence of vertebral height loss. Many procedural factors, such as morphology of cement distribution (solid mass) [6, 7, 10], region of cement distribution (non-cement-endplate-contact) [5, 8], lower volume of cement injected [9, 11] and higher vertebral height restoration [4–8, 10, 11], were found to be significantly associated with vertebral height loss. These results might have been thought to be meaningful to guide management in surgical practice. Also, significant patient-related and radiological factors were discussed.

However, most investigates had token immediate postoperative vertebral height as a baseline to define vertebral height loss in their studies [1, 4–9, 12, 13]. Immediate postoperative was a time point when fracture was unhealed or unstable. Theoretically, the value of immediate postoperative vertebral height was the peak point due to a compensatory height increase of unstable property of fracture [6], as vertebral height increase or decrease could partially contribute to the effect of postual reduction or standing up. Hence, vertebral height loss would have been overestimated on both the occurrence and the degree. Subsequently, significant risk factors of vertebral height loss in literature would have been misleading.

In order to cancel out the bias, we defined vertebral height deterioration (VHD) as the presence of a decrease of vertebral height at follow-up compared to preoperative. The definition of VHD was inherently different from that of vertebral height loss, as their baselines were fundamentally different. This might help to provide further insight into whether or not vertebroplasty can avoid further collapse of cemented vertebrae. The aim of the study was to reveal predictors for VHD in fractured vertebrae operated by PVP, with a specific focus on surgical factors.

## Methods

### Patient population

All patients with OVCFs treated by PVP between April 2016 and September 2018 were retrospectively reviewed. A total of 742 patients were screened during the study period. The study protocol was approved by our institutional review board. The diagnosis of osteoporosis was made by a T-score < -2.5 of bone mineral density (BMD) according to dural-enegry X-ray absorptiometry (DEXA), or by radiographic features [definitive decreased bone density on plain radiographs or computed tomography (CT)], or by clinical findings (fracture due to a minor or no trauma). The diagnosis of vertebral fracture was made by plain radiographs, CT or magnetic resonance imaging (MRI). The inclusion criteria included: (1) one or more OVCFs, (2) 5 or more scores of visual analogue scale (VAS) of focal back pain, (3) level of fracture of T6 or lower, (4) aged 60 years or more, (5) at least 12 months of follow-up. The exclusion criteria included: (1) patients who had previous PVA or other spinal surgery (*n* = 98), (2) incomplete radiologic data (*n* = 86), (3) unilateral vertebroplasty (*n* = 10), (4) malignant vertebral fracture (*n* = 5).

### Surgical procedure and follow-up

All the cases were performed by one of three senior surgeons. Under local anesthesia, a routine transpedicular approach was used bilaterally to perform PVP. Then, polymethylmethacrylate (PMMA) was carefully injected into the fractured vertebra with the fluoroscopic control. The injection was terminated when intraoperative live fluoroscopy images demonstrated progressive symmetrical satisfactory filling of the vertebral body, or when cement leakage was noted. Patients with multiple level fractures were treated using a one-stage procedure.

Radiographs were obtained preoperatively, postoperatively and at follow-up (3, 12 months after procedure, and subsequently per year). Also, a CT scan was completed to identity cement leakage postoperatively.

### Clinical, radiological and surgical evaluation

Clinical data were gathered retrospectively from case notes by one author (Y. D. L.). Clinical data included sex, age, body mass index (BMI), fracture cause, fracture levels, fracture age, follow-up time. Fracture age was classified according to duration of symptom as either acute (< 2 weeks), subacute (2–6 weeks), or chronic (>6 weeks) [14].

Radiological data included fracture location, fracture type, fracture severity, fracture region, cortical disruption, intravertebral cleft, spinal canal compromise, and basivertebral foramen. Fracture type was assessed on lateral radiographs and classified according to Genant et al. as either wedge, biconcave, or crush [15]. According to a method of semiquantitative grading of vertebral fractures, fracture severity was classified according to degree of vertebral body reduction in any (anterior, middle, or posterior) height as mild (20–25%), moderate (26–40%), and severe (>40%) on lateral radiographs [15]. Fracture region was detected by MRI and classified according to a modification of Kanchiku methods into three types: inferior, superior, total [16]. Cortical disruption was defined as evident discontinuation at endplates, or anterior, posterior, lateral wall of vertebral body on MRI or CT [17]. An intravertebral cleft was defined as an intravertebral, abnormal, well-demarcated, linear or cystic hypointensity similar to air on radiography or T1-weighted MRI sequences; an abnormal, well-demarcated, linear or cystic hyperintensity similar to cerebrospinal fluid on STIR sequences [18]. Spinal canal compromise was indicated abnormality of spinal canal area due to intrusion of posterior wall on axial CT [19]. Basivertebral foramen was assessed as presence of triangle or trapezoid shaped foramen at posterior wall on middle-sagittal CT or MRI, or as presence of hemicycle shaped foramen on axial CT [20].

**Fig. 1:**
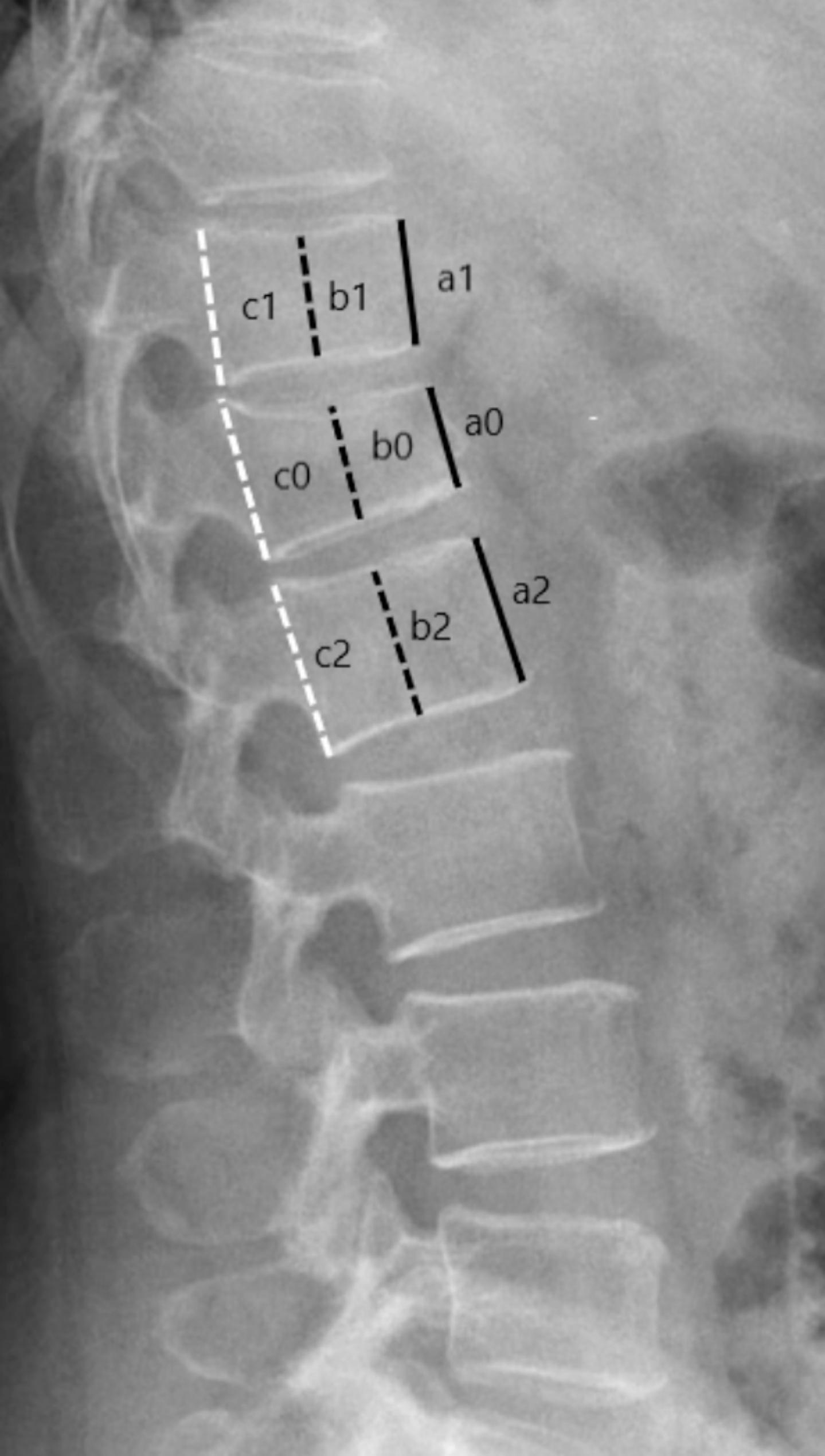
The absolute value of vertebral height is measured at each anterior (**a**), middle (**b**), and posterior (**c**) thirds for one fractured vertebra and two nearest nonfractured vertebrae. For this fractured vertebra, a0 was the smallest among the three value (a0, b0 and c0). Hence, its vertebral height = a0**/[**(a1 + a2)/2]

Surgical data included morphology of cement, region of cement, cement leakage, cement volume, vertebral angle restoration, and vertebral height restoration. Morphology of cement was assessed on lateral radiographs and classified according to Han et al. into two types: interdigitation, when cement was interspersed throughout trabeculae, and solid mass, when cement was lumped without interspersion [21]. Inferior-to-superior region of cement was assessed on lateral radiographs and classified according to a modification of Kim methods into three types: no-endplate contact, one-endplate contact, and two-endplate contact [1]. Lateral-to-lateral region was assessed on frontal radiographs and classified according to He et al. into two types: H-type, when cement was bilaterally discontinuous or partly interdigitated, O-type, when cement was bilaterally continuous and completely interdigitated [22]. Anterior-to-posterior region was assessed on lateral radiographs and classified according to ratio of cement/vertebra anteroposterior dimension as either ≥ 2/3, or<2/3. Any cement leakage was assessed on postoperative CT and classified into 4 types: through basivertebral vein (type B), through segmental vein (type S), through cortical defect (type-C), and intradiscal leakage (type D) [23, 24]. Vertebral angle restoration was calculated as the difference between immediate postoperative and preoperative vertebral angle. Of note, vertebral angle was defined as the angle by two lines passing along fractured vertebra’s endplates [25]. Vertebral height restoration was calculated as the difference between immediate postoperative and preoperative vertebral height. Of note, vertebral height was measured according to Kim et al. at each anterior, middle, and posterior thirds, and then the smallest site one was divided by a mean value of the corresponding cortical heights of the 2 nearest nonfractured vertebrae (Fig. 1) [2].

Radiological and surgical data were collected retrospectively from radiographs, CT and MRI, and evaluated independently by two authors (B. Q. T. and L. B. C.), with discrepancies resolved by a third author (X. M. C.).

### Definition of vertebral height deterioration (VHD)

**Fig. 2:**
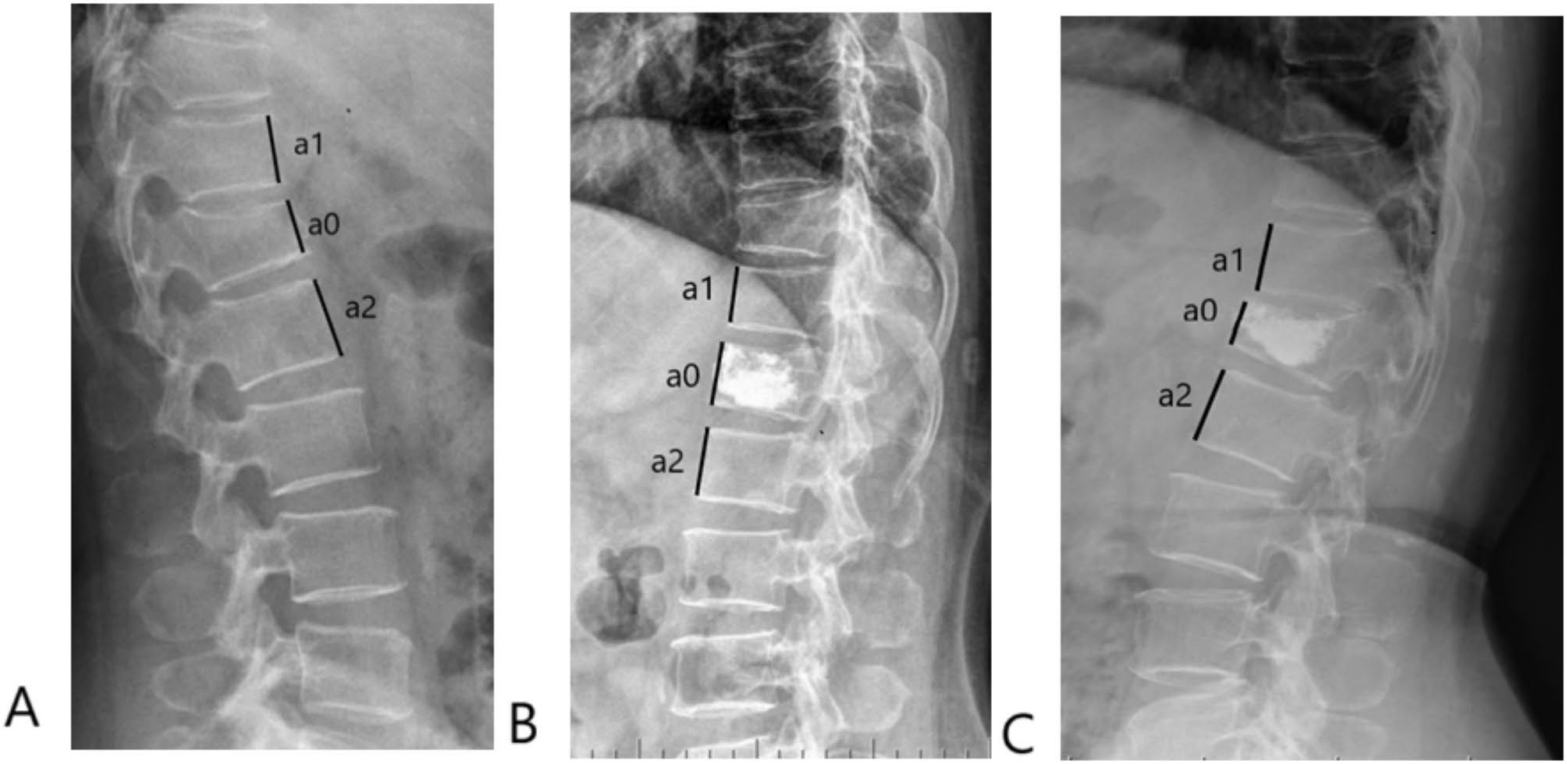
Vertebral height deterioration (VHD) in T12 was observed, as a decrease in vertebral height at final follow-up (**C**) compared to preoperative (**A**). **A** Preoperative lateral radiograph. Preoperative vertebral height = a0/[(a1 + a2)/2] = 0.79. **B** Lateral radiograph at 1-day postoperative time point. Postoperative vertebral height = a0/[(a1 + a2)/2] = 0.95. **C** Lateral radiograph at final follow-up (26-month postoperative time point). Final follow-up vertebral height = a0/[(a1 + a2)/2] = 0.61. In this case, vertebral height deterioration rate (VHDR) = (preoperative vertebral height– final follow-up vertebral height) / preoperative vertebral height × 100% = (0.79–0.61) / 0.79 × 100% = 22.3%

VHD was defined as the presence of a decrease of vertebral height at follow-up compared to preoperative (Fig. 2). On the contrary, vertebral height well-maintained (VHW) was defined as the absence of a decrease or the presence of an increase of vertebral height at follow-up compared to preoperative.

Of note, VHD rate (VHDR) was calculated as: (preoperative vertebral height– final follow-up vertebral height) / preoperative vertebral height × 100% (Fig. 2).

### Statistical analysis

Statistical analysis was performed using the Statistical Package for the Social Sciences (SPSS), V 19.0 (Chicago, IL, USA). Univariate logistic regression models were used to explore effects of clinical, radiological and surgical parameters against occurrence of VHD. Significant correlates at *P* values of less than 0.05 were retained for final multivariate model. Multivariate binary logistic regression model was performed using a stepwise approach to identify independent predictors for VHD. The statistical significance of potential predictors was assessed with the likelihood ratio test. In the final model, a *P* value less than 0.05 was considered significant.

## Results

A total of 543 patients with 681 vertebrae were finally included. Study population was made up of 108 males and 435 females with a mean age of 73.2 ± 8.1 years (range, 60–95 years) (Table 1). Mean follow-up time was 28.9 ± 13.4 months (range, 12–59 months). Clinical, radiological and surgical features were documented (Tables 1, 2 and 3).

**Table 1:** Clinical features

Factors	
No. of patients	543
Sex	
Male	108
Female	435
Mean age (range), yr	73.2 ± 8.1 (60–95)
BMI	23.7 ± 3.8
Fracture cause	
Non-trauma	167
Trauma	376
Fracture levels	
Single level	436
Multiple level	107
Fracture age	
Acute (<2 weeks)	365
Subacute (2–6 weeks)	127
Chronic (>6weeks)	51
Follow-up time, months	28.9 ± 13.4

BMI body mass index

**Table 2:** Radiological features

Factors	
No. of treated vertebrae	681
Fracture location	
Non-thoracolumbar	245
Thoracolumbar	436
Fracture type	
Wedge	387
Biconcave	75
Crush	219
Fracture severity	
Mild	463
Moderate	153
Severe	65
Fracture region	
Inferior	38
Superior	129
Total	514
Cortical disruption	
No	329
Yes	352
Cortical disruption on anterior wall	
No	506
Yes	175
Cortical disruption on posterior wall	
No	662
Yes	19
Cortical disruption on lateral wall	
No	577
Yes	104
Cortical disruption on endoplate	
No	402
Yes	279
Intravertebral cleft	
No	492
Yes	189
Spinal canal compromise	
No	471
Yes	210
Basivertebral foramen	
No	469
Yes	212

**Fig. 3:**
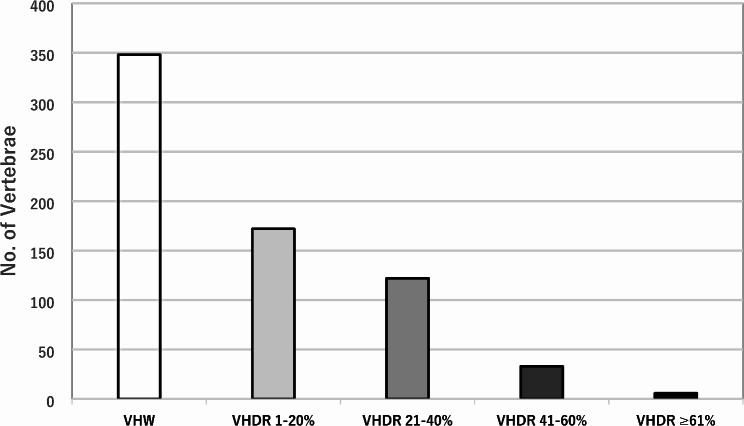
Percentage of vertebrae (*N* = 681) in each group. VHW, vertebral height well-maintained. VHDR, vertebral height deterioration rate

VHD and VHW were found in 48.9% (333/681) and 51.1% (348/681) of all treated vertebrae, respectively. Incidence of VHDR 1–20%, 21–40%, 41–60%, ≥ 61% were 25.3% (172/681), 17.9% (122/681), 4.8% (33/681), 0.9% (6/681), respectively (Fig. 3).

Univariate analysis showed that nine significant factors related to VHD were fracture age, fracture location, fracture severity, cortical disruption on anterior wall, cortical disruption on lateral wall, intravertebral cleft, spinal canal compromise, morphology of cement, and type-C leakage (*p*<0.05). Of note, with regard to surgical factors, region of cement, cement volume, wedge angle restoration, and vertebral height restoration did not reach significance in univariate analysis (Table 4).

**Table 3:** Surgical features

Factors	
No. of treated vertebrae	681
Morphology of bone cement	
Interdigitation	524
Solid mass	157
Region of cement (Inferior-to-superior)	
No-endplate contact	54
One-endplate contact	255
Two-endplate contact	372
Region of cement (Lateral-to-lateral)	
H-type	188
O-type	493
Region of cement (Anterior-to-posterior)	
<2/3	28
≥2/3	653
Cement leakage	
No	188
Yes	493
Type-B leakage	
No	423
Yes	258
Type-S leakage	
No	351
Yes	330
Type-C leakage	
No	622
Yes	59
Type-D leakage	
No	614
Yes	67
Cement volume, ml	5.2 ± 1.8
Wedge angle restoration (°)	4.1 ± 3.7
Vertebral height restoration	0.08 ± 0.06

Multivariate analysis was performed to determine risk factors for VHD as well as their effects. Fracture location, cortical disruption on lateral wall and intravertebral cleft were three risk factors. Thoracolumbar locations exhibited 2.9-fold more at risk for VHD than non-thoracolumbar locations. Cortical disruption on lateral wall and intravertebral cleft exhibited 2.5-fold, 2.4-fold more at risk for VHD, respectively. In contrast, fracture severity and fracture age were two protective factors. For every increase of per grade of fracture severity and fracture age, risk of VHD decreased by 52.1%, 51.3%, respectively. Of note, morphology of cement (OR, 1.476; 95% CI, 0.952–2.287; *p* = 0.082) did not demonstrate its effectiveness to predict VHD in multivariate analysis. Cortical disruption on anterior wall (OR, 1.056; 95% CI, 0.649–1.719; *p* = 0.827), spinal canal compromise (OR, 1.066; 95% CI, 0.716–1.588; *p* = 0.753), type-C leakage (OR, 2.126; 95% CI, 0.979–4.619; *p* = 0.057) did not reach significance in final model either (Table 5).

**Table 4:** Results of univariate analysis for vertebral height deterioration (VHD) in fractured vertebrae

Risk factors	VHD group (*N* = 333)	VHW group (*N* = 348)	OR (95% CI)	*P*
**Clinical factors**				
Sex			0.88 (0.60–1.28)	0.503
Male	69 (20.7%)^*^	65 (18.7%)^*^		
Female	264 (79.3%)^*^	283 (81.3%)^*^		
Mean age (range), yr	73.7 ± 8.3^*^	72.8 ± 7.9^*^	1.02 (1.00-1.03)	0.118
BMI	24.1 ± 4.0^*^	23.7 ± 3.4^*^	1.03 (0.98–1.09)	0.252
Fracture cause			1.08 (0.78–1.50)	0.649
Non-trauma	98 (29.4%)^*^	108 (31.0%)^*^		
Trauma	235 (70.6%)^*^	240 (69.0%)^*^		
Fracture levels			0.84 (0.61–1.15)	0.277
Single level	220 (66.1%)^*^	216 (62.1%)^*^		
Multiple level	113 (33.9%)^*^	132 (37.9%)^*^		
Fracture age			0.55 (0.43–0.71)	0.000
Acute	255 (76.6%)^*^	200 (57.5%)^*^		
Subacute	57 (17.1%)^*^	110 (31.6%)^*^		
Chronic	21 (6.3%)^*^	38 (10.9%)^*^		
Follow-up time, months	28.6 ± 13.7^*^	29.2 ± 13.1^*^	1.00 (0.99–1.01)	0.544
**Radiological factors**				
Fracture location			3.38 (2.42–4.72)	0.000
Non-thoracolumbar	74 (22.2%)	171 (49.1%)		
Thoracolumbar	259 (77.8%)	177 (50.9%)		
Fracture type			1.02 (0.86–1.20)	0.856
Wedge	188 (56.5%)	199 (57.2%)		
Biconcave	37 (11.1%)	38 (10.9%)		
Crush	108 (32.4%)	111 (31.9%)		
Fracture severity			0.67 (0.53–0.84)	0.001
Mild	247 (74.2%)	216 (62.1%)		
Moderate	63 (18.9%)	90 (25.9%)		
Severe	23 (6.9%)	42 (12.1%)		
Fracture region			1.21 (1.00-1.46)	0.056
Superior	55 (16.5%)	74 (21.3%)		
Inferior	15 (4.5%)	23 (6.6%)		
Total	263 (79.0%)	251 (72.1%)		
Cortical disruption			1.18 (0.87–1.59)	0.292
No	154 (46.2%)	175 (50.3%)		
Yes	179 (53.8%)	173 (49.7%)		
Cortical disruption on anterior wall			2.22 (1.56–3.16)	0.000
No	222 (66.7%)	284 (81.6%)		
Yes	111 (33.3%)	64 (18.4%)		
Cortical disruption on posterior wall			2.32 (0.87–6.17)	0.093
No	320 (96.1%)	342 (98.3%)		
Yes	13 (3.9%)	6 (1.7%)		
Cortical disruption on lateral wall			3.03 (1.92–4.77)	0.000
No	259 (77.8%)	318 (91.4%)		
Yes	74 (22.2%)	30 (8.6%)		
Cortical disruption on endoplate			0.86 (0.63–1.16)	0.317
No	203 (61.0%)	199 (57.2%)		
Yes	130 (39.0%)	149 (42.8%)		
Intravertebral cleft			2.67 (1.88–3.79)	0.000
No	208 (62.5%)	284 (81.6%)		
Yes	125 (37.5%)	64 (18.4%)		
Spinal canal compromise			1.66 (1.20–2.31)	0.002
No	212 (63.7%)	259 (74.4%)		
Yes	121 (36.3%)	89 (25.6%)		
Basivertebral foramen			1.22 (0.88–1.69)	0.225
No	222 (66.7%)	247 (71.0%)		
Yes	111 (33.3%)	101 (29.0%)		
**Surgical factors**				
Morphology of cement			1.72 (1.20–2.47)	0.003
Interdigitation	240 (72.1%)	284 (81.6%)		
Solid mass	93 (27.9%)	64 (18.4%)		
Region of cement (Inferior-to-superior)			0.85 (0.67–1.07)	0.168
Non-endplate contact	27 (8.1%)	27 (7.8%)		
One-endplate contact	135 (40.5%)	120 (34.5%)		
Two-endplate contact	171 (51.4%)	201 (57.8%)		
Region of cement (Lateral-to-lateral)			1.38 (0.99–1.94)	0.061
H-type	81 (24.3%)	107 (30.7%)		
O-type	252 (75.7%)	241 (69.3%)		
Region of cement (Anterior-to-posterior)			0.52 (0.24–1.14)	0.102
<2/3	18 (5.4%)	10 (2.9%)		
≥2/3	315 (94.6%)	338 (97.1%)		
Cement leakage			1.38 (0.99–1.94)	0.061
No	81 (24.3%)	107 (30.7%)		
Yes	252 (75.7%)	241 (69.3%)		
Type-B leakage			1.28 (0.94–1.74)	0.120
No	197 (59.2%)	226 (64.9%)		
Yes	136 (40.8%)	122 (35.1%)		
Type-S leakage			0.77 (0.57–1.03)	0.081
No	183 (55.0%)	168 (48.3%)		
Yes	150 (45.0%)	180 (51.7%)		
Type-C leakage			3.73 (2.01–6.93)	0.000
No	288 (86.5%)	334 (96.0%)		
Yes	45 (13.5%)	14 (4.0%)		
Type-D leakage			1.07 (0.61–1.68)	0.951
No	300 (90.1%)	314 (90.2%)		
Yes	33 (9.9%)	34 (9.8%)		
Cement volume, ml	5.3 ± 1.7	5.2 ± 1.8	1.00 (0.92–1.09)	0.919
Wedge angle restoration (°)	4.3 ± 4.0	4.0 ± 3.3	1.02 (0.98–1.07)	0.284
Vertebral height restoration	0.08 ± 0.05	0.07 ± 0.06	2.52 (0.17–37.93)	0.505

^*^Calculated by per vertebra as unit

VHD, vertebral height deterioration; VHW, vertebral height well-maintained; OR odds ratio; CI confidence interval; BMI body mass index

**Table 5:** Results of multivariate analysis for vertebral height deterioration (VHD) in fractured vertebrae

Risk factors	OR (95%CI)	*P*
**Clinical factors**		
Fracture age	0.513 (0.385–0.683)	0.000
**Radiological factors**		
Fracture location (Thoracolumbar)	2.878 (1.994–4.152)	0.000
Fracture severity	0.521 (0.386–0.703)	0.000
Cortical disruption on anterior wall	1.056 (0.649–1.719)	0.827
Cortical disruption on lateral wall	2.535 (1.351–4.758)	0.004
Intravertebral cleft	2.362 (1.488–3.750)	0.000
Spinal canal compromise	1.066 (0.716–1.588)	0.753
**Surgical factors**		
Morphology of cement (Solid mass)	1.476 (0.952–2.287)	0.082
Type-C leakage	2.126 (0.979–4.619)	0.057

VHD, vertebral height deterioration; OR, odds ratio; CI, confidence interval

## Discussion

To our knowledge, qualitatively there were consistent definitions of vertebral height loss, as all the baselines were based on the value of immediate postoperative vertebral height. However, quantitatively the diagnosing criteria of vertebral height loss varied from absolute value of anterior vertebral height decrease ≥ 1 mm [4], absolute value of middle vertebral height decrease ≥ 2 mm [5], absolute value of at least one (anterior, meddle or posterior) vertebral vertebral height decrease ≥ 4 mm [12], absolute value of whole vertebral vertebral height decrease ≥ 4 mm [6], ≥ 15% progression of vertebral compression rate [7, 8], to ≥ 10° progression of local kyphotic angle [7]. In previous PVA series, incidence of vertebral height loss varied from 14.0 to 63.3% [4–8, 12]. In our present study, VHD was defined according to preoperative vertebral height as a baseline. Incidence of VHD in fractured vertebrae operated by PVP was 48.9% (333/681). Of note, only 0.6% (4/681) needed a repeated PVP. Mean follow-up time in present study were comparable to that of previous studies [1, 2, 4–9] and sufficient to capture VHD.

Noticeably, the critical differences on results between our study and previous studies were that with regard to surgical factors there were negative results in our study but positive results in any one of previous studies. It might imply that potential magnitude of overestimation according to previous definition did exist. Hence, this was one of reasons why we specifically focused on surgical factors.

It had been accepted that the reason for vertebral height loss after PVA was multifactorial. Some of clinical or radiological factors were significantly associated with vertebral height loss in previous studies [1, 4–13]. Similarly, in the present study, one clinical and four radiological factors were independent predictors for VHD.

Fracture age was one clinical factor that was significantly associated with VHD. The result was inconsistent with that of many previous studies [1, 5, 6]. One possible explanation was that variation existed between series. For example, predominance of fracture age differed in study population [1, 5, 6].

With respect to radiological parameters, thoracolumbar location was identified to be the strongest risk factor for VHD in the present study. In most multivariate analysis [4–8], thoracolumbar location was not risk factor for vertebral height loss, but it did demonstrate significance in pooled analysis [10]. It makes intuitive sense, as it is the most mobile region where fractures are predisposed to instability or union, it fundamentally increases risk of gradual VHD. Cortical disruption on lateral wall was a second radiological predictor for VHD. Considering that there were negative results on anterior wall, posterior wall and endplates, it might be presumed that an intact lateral wall may pay a key role in maintaining vertebral height for OVCFs patients. Intravertebral cleft was a third radiological predictor for VHD. The results was in line with that of most series [1, 5, 7, 8, 13] and pooled analysis [10, 11]. Plausible explanation may stem from the biomechanical feature of augmented vertebra, given that intravertebral cleft was vulnerable to form unfavorable morphology and insufficient region of cement [1, 5, 7, 8, 10, 11, 13]. Whereas, we speculated that hidden reason may be attributable to pathogenesis of intravertebral cleft itself, as it indicated histological osteonerosis due to avascularity [26, 27]. Given that procedure could not reverse this pathophysiological progress, gradual VHD might not be ceased. Our speculation was similar to that of Heo et al. [13]. Moreover, fracture severity was the fourth radiological factor significantly associated with VHD. The result was not in agreement with that of most series [1, 6, 7, 13]. This was presumed that heterogeneity existed in methodologies. For example, fracture severity had been evaluated by categorical variable as two grades (mild vs. others) [7], or by continuous variable as percentage or ratio [1, 6, 13].

Conversely, with respect to surgical parameters, none was an independent predictor for VDH in our final analysis. It was noted that, in the light of previous literature, one or more surgical parameters had been identified to be significantly associated with vertebral height loss in each study [1, 4–13]. Hence, several factors must be discussed. First, biomechanically, cancellous bone surrounding cement bolus was acted as the weakest link in the chain of force transmission [28]. As solid mass had been regarded as a more pathological distribution way occupying the space [10, 29], it was more likely to crush progressively under repetitive loading conditions [28]. Clinically, solid mass was found to be an independent risk factor for vertebral height loss [6, 7, 10]. Second, biomechanically, two-endplate contact was demonstrated to definitely reduce stress transfer and provide sufficient strengthening in finite element study [30]. Clinically, no-endplate contact was proved to be an independent predictor for vertebral height loss [1, 5, 8]. Third, higher vertebral height restoration and lower volume of cement were reported to be another two significant risk factors for vertebral height loss [4–11, 13]. According to these previous results, surgeons might have attempted to acquire interdigitation of cement in morphology [6, 7, 10], obtain two-endplate contact of cement in region [5, 8], avoid a greater vertebral height restoration [4–8, 10, 11, 13] and inject a higher volume of cement [9, 11], when seeking to reduce the risk of vertebral height loss.

However, all these surgical factors did not reach significance in our final model analysis. One explanation was that confounding factors existed. For example, solid mass was significantly associated with intravertebral cleft [7, 8, 12, 13]. More importantly, the causality would be that using the definition of VHD in analysis had canceled out the bias on vertebral height change, as the change was partially attributable to cement volumetric effect as well as postural reduction during procedure. Parsimonious model selection probably did not exclude important factors in final analysis. In addition, a total of 681 vertebrae were enrolled and 31 parameters were evaluated, making it sufficiently large and comprehensive to allow statistically valid conclusions to be drawn. To some degree, results in our study implied that surgeon’s manipulating surgical factors to avoid the occurrence of VHD might not matter.

Whereas, results should be cautiously explained under certain circumstances. For example, mean volume of cement injected was 5.2 ± 1.8 ml, or follow-up time was at least 12 months in this series. Still, it should be emphasized that surgical parameters were crucial for each case in practice, as they were closely related to clinical and radiological outcomes. Of note, vertebroplasty had showed better results on vertebral height at long-term follow-up compared to conservative treatment in previous study [25].

Our study adds valuable new information to the literature and could guide high quality future studies on this issue. The definition of VHD was proposed in analysis, providing a better understanding of its incidence and risk factors. At least, we suggested that surgeons should not overestimate the long-term effect on vertebral height maintenance from vertebroplasty.

Several limitations must also be acknowledged. First and foremost, there was inherent limitation of retrospective design, such as potential selection bias and data completeness issues. Hence, our results should not be overstated beyond the methodology of this study. Second, classification of fracture age may be primitive, as it was difficult to record accurate duration of symptoms in aged patients who had memory impairment. Third, many severe fractures were excluded, as they were usually considered as candidates for PKP or spinal reconstructive surgery at our center. Third, we did not include kyphotic angle restoration [6–8, 11] in analysis. Kyphotic angle was more primitive than vertebral angle, as the former contains one additional vertical disc and normal vertebra, which biased effect of procedure. Fourth, comorbidities, T-score of BMD / severity of osteoporosis, and medication for osteoporosis were not evaluated, which might bias the results. Fifth, we could not evaluate the relationship between VHD and pain relief, as this was beyond the landscape of current study. Sixth, the study was conducted at a single center in China, and the results may not be applicable to other populations with different demographics. Seventh, there was lack of a control group for conservative treatment or internal fixation. The clinical meaning of VHD needs to explore in further studies.

## Conclusions

VHD in fractured vertebrae operated by PVP might be inevitable, as it was prevalent (48.9%, 333/681). One clinical factor and four radiological factors, including fracture age, fracture location, cortical defect on lateral wall, intravertebral cleft and fracture severity, were significant predictors for VHD. However, none of surgical factors reached significance in final analysis. The results implied that surgical factors might play a negligible effect on HVD. VHD might be due to natural course of fracture/osteoporosis.

## Data Availability

The datasets used and analysed during the current study are available from the corresponding author on reasonable request.

## Abbreviations

PVA: Percutaneous vertebral augmentation
OVCFs: Osteoporotic vertebral compression fractures
VHD: Vertebral height deterioration
PVP: Percutaneous vertebroplasty
PKP: Percutaneous kyphoplasty
BMD: Bone mineral density
DEXA: Dural-enegry X-ray absorptiometry
CT: Computed tomography
MRI: Magnetic resonance imaging
VAS: Visual analogue scale
PMMA: Polymethylmethacrylate
BMI: Body mass index
VHDR: Vertebral height deterioration rate
SPSS: Statistical package for the social sciences
